# Ovarian cancer cells direct monocyte differentiation through a non-canonical pathway

**DOI:** 10.1186/s12885-020-07513-w

**Published:** 2020-10-17

**Authors:** Kaitlin C. Fogg, Andrew E. Miller, Ying Li, Will Flanigan, Alyssa Walker, Andrea O’Shea, Christina Kendziorski, Pamela K. Kreeger

**Affiliations:** 1 Department of Biomedical Engineering, University of Wisconsin-Madison, 1111 Highland Ave, WIMR 4553, Madison, WI 53705 USA; 2 Department of Biostatistics and Medical Informatics, University of Wisconsin School of Medicine and Public Health, Madison, WI USA; 3 Department of Obstetrics and Gynecology, University of Wisconsin School of Medicine and Public Health, Madison, WI USA; 4 Department of Cell and Regenerative Biology, University of Wisconsin School of Medicine and Public Health, Madison, WI USA; 5 University of Wisconsin Carbone Cancer Center, University of Wisconsin School of Medicine and Public Health, Madison, WI USA

**Keywords:** M2 macrophage, Tumor associated macrophage, EGFR, mAb225

## Abstract

**Background:**

Alternatively-activated macrophages (AAMs), an anti-inflammatory macrophage subpopulation, have been implicated in the progression of high grade serous ovarian carcinoma (HGSOC). Increased levels of AAMs are correlated with poor HGSOC survival rates, and AAMs increase the attachment and spread of HGSOC cells in vitro. However, the mechanism by which monocytes in the HGSOC tumor microenvironment are differentiated and polarized to AAMs remains unknown.

**Methods:**

Using an in vitro co-culture device, we cultured naïve, primary human monocytes with a panel of five HGSOC cell lines over the course of 7 days. An empirical Bayesian statistical method, EBSeq, was used to couple RNA-seq with observed monocyte-derived cell phenotype to explore which HGSOC-derived soluble factors supported differentiation to CD68+ macrophages and subsequent polarization towards CD163+ AAMs. Pathways of interest were interrogated using small molecule inhibitors, neutralizing antibodies, and CRISPR knockout cell lines.

**Results:**

HGSOC cell lines displayed a wide range of abilities to generate AAMs from naïve monocytes. Much of this variation appeared to result from differential ability to generate CD68+ macrophages, as most CD68+ cells were also CD163+. Differences in tumor cell potential to generate macrophages was not due to a MCSF-dependent mechanism, nor variance in established pro-AAM factors. TGFα was implicated as a potential signaling molecule produced by tumor cells that could induce macrophage differentiation, which was validated using a CRISPR knockout of *TGFA* in the OVCAR5 cell line.

**Conclusions:**

HGSOC production of TGFα drives monocytes to differentiate into macrophages, representing a central arm of the mechanism by which AAMs are generated in the tumor microenvironment.

## Background

High grade serous ovarian carcinoma (HGSOC) is the deadliest gynecological malignancy, accounting for 70% of all ovarian cancer related mortalities [1]. The late-stage of diagnosis, coupled with extensive metastasis to secondary sites, renders the five-year survival rate of HGSOC at less than 30% [2]. The tumor microenvironment of HGSOC is highly complex, comprising multiple different types of cells as well as a diverse range of chemical and physical cues [3, 4]. A key cell type present in most solid tumors is a macrophage, accounting for up to 40% of tumor mass; increased macrophage numbers correlate with poor patient outcomes across many cancer types [5]. Macrophages are vital members of the innate immune system, playing roles in both initiation and resolution of the inflammatory response [6]. This duality of function is primarily due to the plasticity of macrophages, which exist along a spectrum ranging from classically-activated macrophages (CAMs, pro-inflammatory) to alternatively-activated macrophages (AAMs, anti-inflammatory). Both CAMs and AAMs are derived from monocytes, which infiltrate wound beds or tumor microenvironments in response to secretion of chemokine ligand 2 (CCL2, also known as MCP-1). Once monocytes differentiate to macrophages, they continuously interpret microenvironmental cues and respond by adjusting their polarization, altering their position along the inflammatory spectrum accordingly [7–9].

Many tumor-associated macrophages have a pro-tumor, anti-inflammatory phenotype comparable to alternatively-activated macrophages [10, 11]. In HGSOC, elevated levels of macrophages with this alternative polarization in the ascites fluid correlate with poor patient survival [12–14]. In vivo, a resident population of omental CD163+ macrophages has been shown to be essential for metastatic spread in a mouse model of ovarian cancer [15]. Using in vitro models of HGSOC, we have previously demonstrated that AAMs secrete soluble factors such as MIP-1β, HB-EGF, and leptin, resulting in increased ovarian cancer cell adhesion, proliferation, and spreading, respectively [16–18]. In these studies, AAMs were generated by differentiating naïve monocytes into macrophages with MCSF and then polarizing towards the alternative phenotype through IL-4 and IL-13 treatment, resulting in a cell population that is CD68+, CD163+ [19]. However, while the pro-tumorigenic influence of AAMs on ovarian cancer progression has been established, the reciprocal question of the role of the tumor cell in the generation of AAMs from monocytes has not been well characterized in HGSOC.

Identifying mechanisms by which monocytes are differentiated to macrophages, polarized towards an AAM phenotype, and then stabilized in that phenotype could potentially identify new targets to control cancer progression. Prior studies have demonstrated that different tumor subtypes utilized distinct cancer-macrophage signaling axes [10]; thus, we hypothesized that ovarian cancer cells may produce a unique subset of soluble factors to regulate this process. To explore this hypothesis, we modified an in vitro co-culture model previously developed in our lab [20] to include naïve primary human monocytes and five human ovarian cancer cell lines. We then used this system to examine the macrophage differentiation/polarization potential of ovarian cancer cell lines. Based on our observations and the results of RNA-seq of the selected cancer cell lines, we then use bioinformatics approaches and experimental follow-up to identify potential mechanisms involved in ovarian cancer cell direction of macrophage differentiation.

## Methods

Unless stated, all reagents were purchased from ThermoFisher (Waltham, MA).

### Monocyte isolation and HGSOC culture

Whole blood from healthy females (age 18–55) was purchased from Zen Bio (Durham, NC). Monocytes were enriched using RosetteSep Human Monocyte Enrichment Cocktail in combination with SepMate 50 mL tubes according to the manufacturer’s instructions (STEMCELL Technologies, Seattle, WA). Human HGSOC cell lines OV90 and OVCAR3 were purchased from ATCC (Manassas, Virginia), OVCA432 and OVCAR5 were obtained from Dr. R. Bast (MD Anderson Cancer Center; Houston, TX, USA), and OVCAR4 were obtained from the NCI Tumor Repository (Frederick, MD). All ovarian cancer cell lines were maintained in ovarian cancer media (1:1 (v/v) ratio of MCDB105: Medium199 (Corning, Corning, NY) supplemented with 1% penicillin/streptomycin) with 15% heat-inactivated fetal bovine serum prior to co-culture.

### In vitro micro-culture device

For all co-culture studies, an in vitro micro-culture device was used that allows for the examination of paracrine signaling in a controlled environment [20]. The microdevice was constructed by placing an oval PDMS ring (11 × 17 × 0.5 mm) in a well of a 24 well plate. Freshly isolated monocytes were seeded in each well at a concentration of 400,000 cells per well in 40 μL of AIMV media supplemented with 1% penicillin-streptomycin. Glass coverslips (9 × 9 mm) were coated with 8 μg/mL collagen I (Sigma, St. Louis, MO) in PBS overnight, after which point ovarian cancer cells were dissociated with trypsin and seeded on the coverslips at a concentration of 25,000 cells per coverslip in ovarian cancer media.

Co-culture was initiated by inverting the ovarian cancer-containing coverslip and placing it on top of the PDMS ring that contained either naïve monocytes or AIMV alone. Every 2 days a new HGSOC coverslip was added to the co-culture device and the AIMV media was replaced, and 4 μL of AIMV media was added to each device on alternate days to counteract evaporation. For some experiments, HGSOC cells were cultured on coverslips in the device without monocytes. AAM control monocultures were generated as previously described [16]. Briefly, monocytes were differentiated into macrophages over the course of 5 days in AIMV media supplemented with 1% penicillin–streptomycin and 20 ng/mL MCSF. Macrophages were subsequently polarized to AAMs over the course of 2 days in AIMV media supplemented with 1% penicillin–streptomycin and 2 ng/mL IL-4 and IL-13. To generate CAMs monocytes were differentiated into macrophages over the course of 5 days in AIMV media supplemented with 1% penicillin–streptomycin and 20 ng/mL GMCSF, then polarized to CAMs by culturing the cells in AIMV media supplemented with 1% penicillin–streptomycin, 20 ng/mL IFNγ and 100 ng/mL lipopolysaccharide (LPS) [21].

### Immunocytochemistry

Following 7 days of co-culture, cancer cell coverslips were removed from the top of the PDMS ring and the monocyte derived cells (MDCs) at the bottom of the microdevice were fixed for 30 min using 4% paraformaldehyde. MDCs were rinsed with 0.1% BSA in PBS then permeabilized and blocked in buffer containing 0.3% Triton X-100 and 1% normal goat serum in PBS for 45 min at room temperature. Cells were then incubated with primary antibodies diluted in dilution buffer containing 1% BSA, 0.3% Triton-X, 1% normal goat serum, and 0.01% sodium azide for 2 h at 25 °C [anti-CD68 (1:200, ab213363, Abcam, Eugene, OR), anti-CD163 (1:100, ab182422, Abcam)]. After rinsing with PBS, all cells were incubated with secondary antibody diluted in the same dilution buffer as the primary antibodies (1:500 goat anti-rabbit IgG Alexa Fluor 488) for 1 h at room temperature protected from light, then counterstained with Hoescht 33,258 for 5 min (1:1000). All images were obtained using a Zeiss Axio Observer.Z1 inverted microscope with an AxioCam 506 mono camera, Plan-Apochromat 20 × 0.16-NA air objective, and Zen 2 software (Zeiss). ImageJ software (NIH) was used to quantify CD68+ and CD163+ counts per field of view, and percent CD68 and CD163 were calculated as the ratio of CD68+ or CD163+ counts to total cell counts from the Hoechst signal.

### RT-qPCR analysis of gene expression

RNA was isolated from HGSOC cells in monoculture or co-culture at day 7 using the RNeasy Mini Kit (Qiagen, Germantown, Maryland) according to the manufacturer’s instructions. cDNA was synthesized and amplified using the RT^2^ PreAMP cDNA Synthesis Kit, then assayed using a custom RT^2^ Profiler PCR array for *IL4, IL10, CSF1, CCL2, IL13, TNF,* and *IFNG* (Qiagen) in a CFX real-time PCR machine (Bio-Rad, Pleasanton, CA) for a total of 40 cycles, using Qiagen’s Data Analysis Center for analysis. Data are expressed as fold change, with ±2-fold set as the threshold for significance.

### Bioplex analysis of cytokine profiles

Conditioned media was collected from HGSOC cells in monoculture or co-culture at day 7. Media samples were centrifuged at 10,000 g for 10 min, and the supernatant was frozen at − 80 °C until assayed. Quantification of cytokines IL-15, VEGF, IL-9, PDGF-BB, IL-5, MIP-1β, RANTES, GCSF, IL-12, IL-13, IL-7, IL 17, IL-1ra, bFGF within the conditioned media was performed using a Human Cytokine Th1/Th2 Assay (Bio-Rad). Protein quantification was performed using a MAGPIX instrument (Luminex Corporation, Madison, WI) and assessed using xPONENT for MAGPIX software.

### RNA-seq

RNA was isolated from four biological replicates each of the five HGSOC cell lines using the RNeasy Mini Kit (Qiagen) according to the manufacturer’s instructions. RNA quality was assessed at the University of Wisconsin Biotechnology Center using an Agilent RNA PicoChip and sample libraries were prepared using poly-A selection with TruSeq Stranded mRNA Library Prep Kit (Illumina, Madison, WI) according to manufacturer’s instructions. Prepared libraries were sequenced on an Illumina HiSeq 2500 targeting a read depth of 25 million reads per sample by the University of Wisconsin Bioinformatics Resource Center.

### Data processing and differential expression analysis

Paired end sequencing (2 × 125 bases) was performed on each sample in one lane of a Illumina HiSeq 2500 sequencer. Reads were mapped back to the genome using the short read aligner Bowtie followed by RSEM [22] to estimate gene expression. All samples passed quality-control and were used in downstream analyses, which were carried out in R (R Development Core Team, 2012). Specific software packages were obtained from Bioconductor, an online suite of tools for the analysis of genomic data [23] unless otherwise noted. DESeq2 [24] was used to visualize the individual gene expression contrasts between the OV90 cell line and OVCAR3, OVCAR4, OVCAR5, and OVCA432 cell lines, as well as to perform principal component analysis. Heatmaps of Pearson correlations between all samples were synthesized and visualized with the package pheatmap v1.0.12 [25]. Differential expression data from DESeq2 outputs were visualized in Venn diagram form using the Limma package [26]

EBSeq [22] was applied to identify differentially expressed genes. To determine if a gene is differentially expressed, we considered six potential patterns of expression (P1-P6, below) across our five cell lines. For each gene, the normalized read counts in condition 1 (*μ*1: OV90) were compared with that in condition 2 (*μ*2: OVCAR4), condition 3 (*μ*3: OVCAR3), condition 4 (*μ*4: OVCAR5) and condition 5 (*μ*5: OVCA432).

$$ P1:\mu 1\ne \mu 2\ne \mu 3\ne \mu 4\ne \mu 5 $$

$$ P2:\mu 1=\mu 2\ne \mu 3\ne \mu 4\ne \mu 5 $$

$$ P3:\mu 1\ne \mu 2=\mu 3\ne \mu 4\ne \mu 5 $$

$$ P4:\mu 1\ne \mu 2\ne \mu 3=\mu 4\ne \mu 5 $$

$$ P5:\mu 1\ne \mu 2\ne \mu 3\ne \mu 4=\mu 5 $$

$$ P6:\mu 1=\mu 2=\mu 3\ne \mu 4=\mu 5 $$

EBSeq calculates the posterior probability of a gene being in each expression pattern. False discovery rates were controlled within EBSeq at 5%. To further sort the genes into those patterns that correlated with observed pro-AAM potential, gene expression was further evaluated for those that adhered to one of the two criteria:

$$ C1:\mu 1\le \mu 2\le \mu 3\le \mu 4\le \mu 5 $$

$$ C2:\mu 1\le \mu 2\le \mu 3\le \mu 4\ge \mu 5 $$

This resulted in two final expression patterns that mimicked the number of CD68+ and CD163+ cells in co-culture (Supplemental Figure 1).

### Inhibition of candidate factor signaling

Inhibition of signaling molecules identified using RNA-seq was performed by supplementing AIM-V media with small molecule inhibitors or antibodies in OVCAR5 co-cultures. FAM3C was inhibited with 10 μM DAPT [27], a γ-secretase inhibitor that blocks FAM3C signaling pathway activation; LIF and TGFα downstream signaling was inhibited using 5 μM galiellalactone (GL), a selective STAT3 inhibitor [28]; AMH signaling was inhibited via the addition of 120 ng/mL anti-AMHR2 according to manufacturer’s instructions (Abcam); the receptor for TGFα and epigen (EGFR) was inhibited with 10 μg/mL mAb225 (Abcam) [17]. The vehicle control medium was AIMV supplemented with 10 μg/mL IgG1 isotype control [17]. 1X concentrations of inhibitors were replaced in 40 μL AIMV media every other day, and 10X concentrations of inhibitors were added in 4 μL AIMV media on alternate days to counteract evaporation.

### CRISPR mediated knockout of TGFA and LIF in OVCAR5 cells

CRISPR/Cas9 was used to knock out *TGFA* or *LIF* in OVCAR5 cells. Briefly, a 20 oligonucleotide (oligo) guide RNA (gRNA) targeting either the *TGFA* or *LIF* loci was designed using CHOPCHOP to minimize off-target binding [29]. The gRNA sequences (*TGFA* gRNA oligos: FWD 5′–CACCGGTGCACCAACGTACCCAGAA–3′, REV 5′–AAACTTCTGGGTACGTTGGTGCACC–3′; *LIF* gRNA oligos: FWD 5′–CACCGGCGGGAAGTCCGTCACGTTG–3′, REV 5′–AAACCAACGTGACGGACTTCCCGCC–3′) were flanked with the NGG PAM sequence on the 3′ end and were synthesized by Integrated DNA Technologies (Coralville, IA). lentiCRISPR v2 was a gift from Feng Zhang (Addgene plasmid #52961; http://n2t.net/addgene:52961; RRID:Addgene_52,961); this one vector system delivers hSpCas9 and chimeric gRNA expression cassettes [30]. The lentiCRISPRv2 vector was dephosphorylated and digested with *BsmBI* restriction enzyme and subsequently gel-purified. The forward and reverse gRNA oligos were phosphorylated, annealed, and ligated into the cut vector. One Shot Stbl3 Chemically Competent *E. coli* (C737303, Invitrogen) were transformed with the *TGFA* or *LIF* lentiCRISPRv2 plasmid and grown in LB with 100 μg/mL ampicillin for selection and expansion. HEK293T cells were co-transfected with packaging plasmids from the Lenti-vpak Lentiviral Packaging Kit (TR30037, Origene) and either the *TGFA or LIF* lentiCRISPRv2 plasmid, using MegaTran 1.0 as the transfection agent. After 48 and 72 h of incubation, viral batches were collected and combined for each gene target. This viral supernatant was used to deliver the *TGFA*- or *LIF*-targeting CRISPR/Cas9 system into the OVCAR5 cell line at a 2:3 virus to ovarian cancer media volume ratio. Polybrene was added at 8 μg/mL, and the plate was centrifuged at 165 g for 2 h to aid transduction. After incubation for 48 h, 10 μg/mL puromycin (A1113803, Invitrogen) was added for an additional 48 h for selection. To confirm successful cutting at the *TGFA* or *LIF* loci, genomic DNA was isolated for Sanger Sequencing with the following primers (*TGFA* FWD 5′–TGTCATGAACACATGTGCTGCC–3′, REV 5′–TTCTGGTGCTGATGGAAGGAGG–3′; *LIF* FWD 5′–GGCTAGACACCGAGTTTTCCCT–3′, REV 5′– CCTGAGATCCCTCGGTTCACAG–3′). Sequencing was performed by the UW-Madison Biotechnology Center. The mutant *TGFA* and *LIF* sequences were aligned to WT *TGFA* and WT *LIF* sequences respectively, using Tracking of Indels by DEcomposition (TIDE) software [31]. These cell lines were termed OVCAR5^TGFA−/−^ and OVCAR5^LIF−/−^. To confirm a knockout on the protein level, an ELISA for TGFα and LIF (R&D Systems, Minneapolis, MN) was performed according to manufacturer’s instructions.

### Statistical analysis

Data are presented as mean ± standard deviation of the mean. Statistical analysis (one-way ANOVA, two-way ANOVA, or t-tests when appropriate) was performed in Prism 7 software (Graph-Pad, San Diego, CA).

## Results

### HGSOC cell lines promote primary monocyte differentiation and polarization towards AAM phenotype

**Fig. 1:**
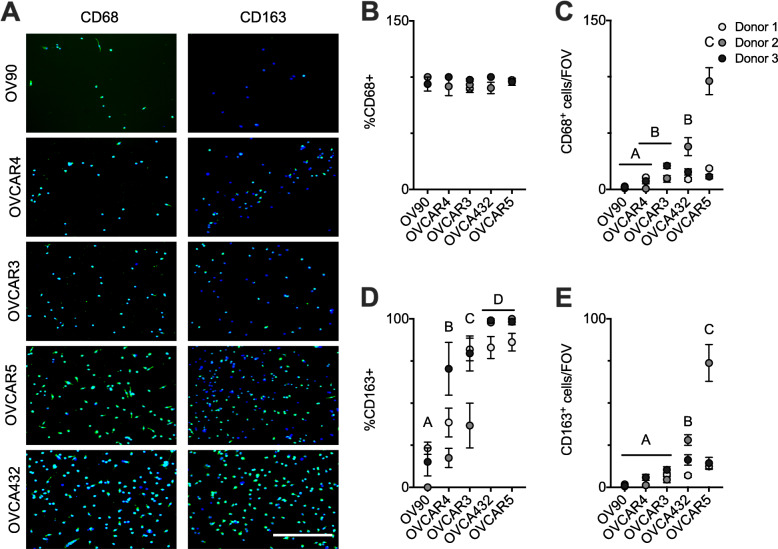
Co-culture with HGSOC cell lines promoted the differentiation and polarization of primary human macrophages towards the alternatively activated macrophage (AAM) phenotype. **a** Immunofluorescent detection of CD68 (a macrophage marker, green) and CD163 (an AAM marker, green) in monocytes that have been co-cultured with HGSOC cell lines for 7 days. Cells were counterstained with DAPI (blue). Scale bar = 250 μm. **b**-**e** Percentage of cells that were CD68+ (**b**), number of CD68+ cells per field of view (**C**), percentage of cells that were CD163+ (**d**), and number of CD163+ cells per field of view (**e**) after monocytes have been co-cultured with HGSOC cell lines for 7 days. Data are expressed as average ± SD, *n* = 3 monocyte donors. Groups with no statistically significant differences are linked by the same letters, while groups that are statistically different from each other have different letters, *p* < 0.05 by Tukey-HSD

We co-cultured primary human monocytes from three unique donors with a panel of five HGSOC cell lines (OV90, OVCAR4, OVCAR3, OVCA432, and OVCAR5) that have been categorized as genomically-consistent with patient samples [32]. Monocytes are non-adherent; therefore, by washing the culture prior to fixing and staining any remaining monocytes are removed. The adherent monocyte derived cells (MDCs) are expected to be either macrophages (CD68+) or dendritic cells (CD68-) [33]. In the experimental condition where monocytes were neither co-cultured with HGSOC cells nor additional serum/cytokines/growth factors very few MDCs were observed (data not shown). In contrast, published classical and alternative activation protocols resulted in robust production of CD68+ macrophages, with the classical activation resulting in CD163- cells and the alternative activation resulting in CD163+ for all three monocyte donors (Supplemental Figure 2). Co-culture with all five lines tested resulted in MDCs; immunostaining for CD68 (Fig. 1a) confirmed that nearly all MDCs were macrophages (Fig. 1b). This is similar to prior reports where in vitro differentiation to dendritic cells by GM-CSF/IL-4 was inhibited by soluble factors secreted by lung cancer cells [34], and suggests that monocyte differentiation in tumors is skewed towards CD68+ macrophages. While all co-cultures resulted in the production of CD68+ macrophages, the number varied considerably across the five HGSOC cell lines (Fig. 1c), consistent with the heterogeneity observed in ovarian cancer [32, 35]. When co-cultured with OV90 or OVCAR4, very few MDCs remained in the micro-culture device. However, when co-cultured with OVCAR3 or OVCA432, significantly more MDCs remained, and co-culture with OVCAR5 yielded the most macrophages. As the co-culture device prevents physical contact between the two cell types [20], we hypothesized that HGSOC cells were driving macrophage differentiation through soluble cues.

Given that co-culture induced macrophage differentiation, we next assessed the ability of co-culture to impact polarization to AAMs by immunostaining for CD163, an AAM specific marker (Fig. 1a). Co-culture resulted in polarization into AAMs, again with variation observed across the five HGSOC cell lines (Fig. 1d). However, here the trends were slightly different. While nearly all MDCs were CD68+ for all cell lines, the percentage of CD163+ cells differed across the tumor lines. Specifically, few of the macrophages from co-culture with OV90 polarized towards an AAM phenotype, significantly more polarized towards AAMs when co-cultured with OVCAR4, even more with OVCAR3, and co-culture with OVCAR5 and OVCA432 yielded the highest percent of CD163+ cells [36]. Of course, to develop a CD163+ cell, the monocyte must first differentiate to a macrophage and then polarize; therefore, the number of CD163+ cells is a function of both the ability to differentiate and the ability to polarize. However, rather than seeing co-cultures that produced numbers of CD163+ cells that were incongruous with the number of CD68+ cells/percentage of CD163+, we observed a similar trend between CD68+ and CD163+ rankings (Fig. 1c, e). This suggests that the largest barrier to producing an AAM may be the differentiation of a monocyte to a macrophage, rather than the polarization. While macrophages can be highly plastic in terms of phenotype there is little evidence that they dedifferentiate back into monocytes [9]. Overall, while there was monocyte donor-to-donor variability, the five HGSOC cell lines consistently exhibited either a lower or higher ability to produce CD68+ macrophages from monocytes and CD163+ AAMs from macrophages. Therefore, to determine the pro-AAM potential of each cell line, we consider both the number of CD68+ cells and the number of CD163+ cells.

### Pro-AAM potential of HGSOC cell lines does not correlate with the secretion of established macrophage differentiation and polarization factors

**Fig. 2:**
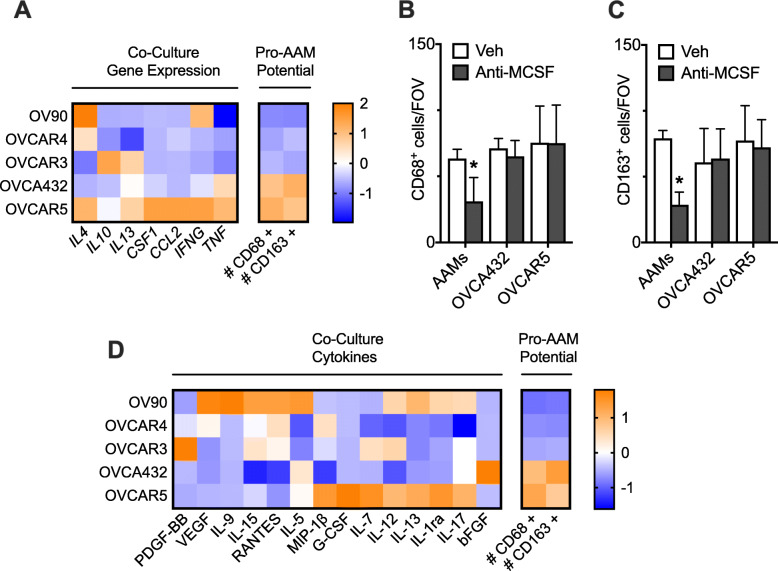
The pro-AAM potential of the HGSOC cell lines was not correlated with the secretion of established pro-AAM factors. **a** Gene expression (Z-score normalized) from HGSOC cell lines in co-culture compared to their pro-AAM potential (the resulting number of CD68+ and CD163+ cells after 7 days of co-culture). Values represent the average of 4 microdevices. **b** CD68+ per field of view and (**c**) 163+ cells per field of view after monocytes have been alternatively activated using MCSF, IL4, and IL13 (AAMs) or co-cultured with HGSOC cell lines OVCAR5 and OVCA432 for 7 days, +/− an anti-MCSF antibody. Data are expressed as average ± SD, *n* = 4 microdevices per condition; *p < 0.05 vs. Veh. by two-way ANOVA with Tukey multiple comparison correction. **d** Cytokine expression (Z-score normalized) from HGSOC cell lines in co-culture compared to their pro-AAM potential. Values represent the average of 4 microdevices

To determine which HGSOC-derived factors were responsible for the generation of macrophages/AAMs, we examined gene expression from the HGSOC cells that had been co-cultured with monocytes/MDCs using a PCR array for factors known to recruit monocytes (*CCL2*), differentiate monocytes into macrophages (*CSF1*), polarize macrophages into AAMs (*IL4, IL10, IL13*), or polarize macrophages into CAMS (*TNF*, *IFNG*) (Fig. 2a). Similar expression levels were seen from HGSOC cells in monoculture or co-culture with monocytes, indicating minimal reciprocal effects from the monocytes on these pathways (Supplemental Figure 3). We did not observe an obvious correlation of gene expression of any factor with the relative CD68+/CD163+ production. MCSF, IL-4, and IL-13 are accepted as the canonical pathway that act in concert to differentiate monocytes into macrophages and subsequently polarize macrophages into AAMs [37, 38]. Intriguingly, the two cell types that produced the most CD68+/CD163+ macrophages, OVCAR5 and OVCA432, demonstrated very different gene expression patterns of the canonical CSF1, IL4, and IL3 cytokines. As our dominant effect of co-culture was the production of CD68+ macrophages, we next directly tested the impact of blocking the canonical MCSF pathway in co-culture. While neutralizing MSCF significantly decreased the number of CD68+ cells produced from monocytes treated with exogenous MCSF, IL-4, and IL-13, it did not significantly impact the ability of CD68+ macrophages to form in co-culture with OVCAR5 and OVCA432 (Fig. 2b). Similar to the trend of differentiation being the limiting step in unperturbed co-culture, the level of CD163+ AAMs was inhibited in the canonical treatment condition plus anti-MCSF, but was unimpacted with MCSF neutralization in co-culture (Fig. 2c). This is in contrast to prior studies demonstrating that attenuation of MCSF signaling through CSF-1R inhibition in glioma led to AAM re-education towards a pro-inflammatory M1-like phenotype [39].

As it is possible that other established pathways are responsible for the observed effects of co-culture, we next examined conditioned media collected from co-cultures to test for additional soluble factors that have been reported to influence macrophage polarization or differentiation (Fig. 2d). All 14 cytokines included in the screen were detected, with variability noted across HGSOC cell lines. Similar to the gene expression data for the canonical factors, OVCAR5 and OVCA432 co-cultures had different patterns of cytokine production that did not correlate with macrophage or AAM production. We also considered whether an increase in CAM-associated cytokines could be responsible for the lower pro-AAM potential in the other three lines, as pro-inflammatory cytokines such as GM-CSF, interferon-gamma (IFN-γ), and IL-12 can skew macrophage populations towards the CAM phenotype and away from an AAM phenotype [39, 40]. When we examined for univariate correlations between either gene expression levels or soluble factors and the gradient of macrophage or AAM production potential across the five HGSOC cell lines in co-culture or monoculture, the strongest correlation was for *TNF* with an R^2^ value of 0.73 (Supplemental Figure 3). However, this was a positive correlation and prior reports have demonstrated that *TNF* induces the CAM phenotype instead of the AAM phenotype [41]. Since the levels tested here reflect the production of cytokines from MDCs as well as HGSOC cells, we also tested the levels from HGSOC in monoculture (Supplemental Figure 3). While the levels of cytokines were different than in co-culture, we did not observe a correlation between any single factor and macrophage or AAM production. We also examined the potential for a multivariate relationship using partial least squares regression [42], but no such relationship was identified (data not shown). Taken together, these data suggest that HGSOC cells were not using established macrophage differentiation or AAM polarization factors and may instead utilize a non-canonical signaling mechanism.

### RNA-seq analysis of HGSOC cell lines coupled with EBSeq identifies potential drivers of AAM-potential

**Fig. 3:**
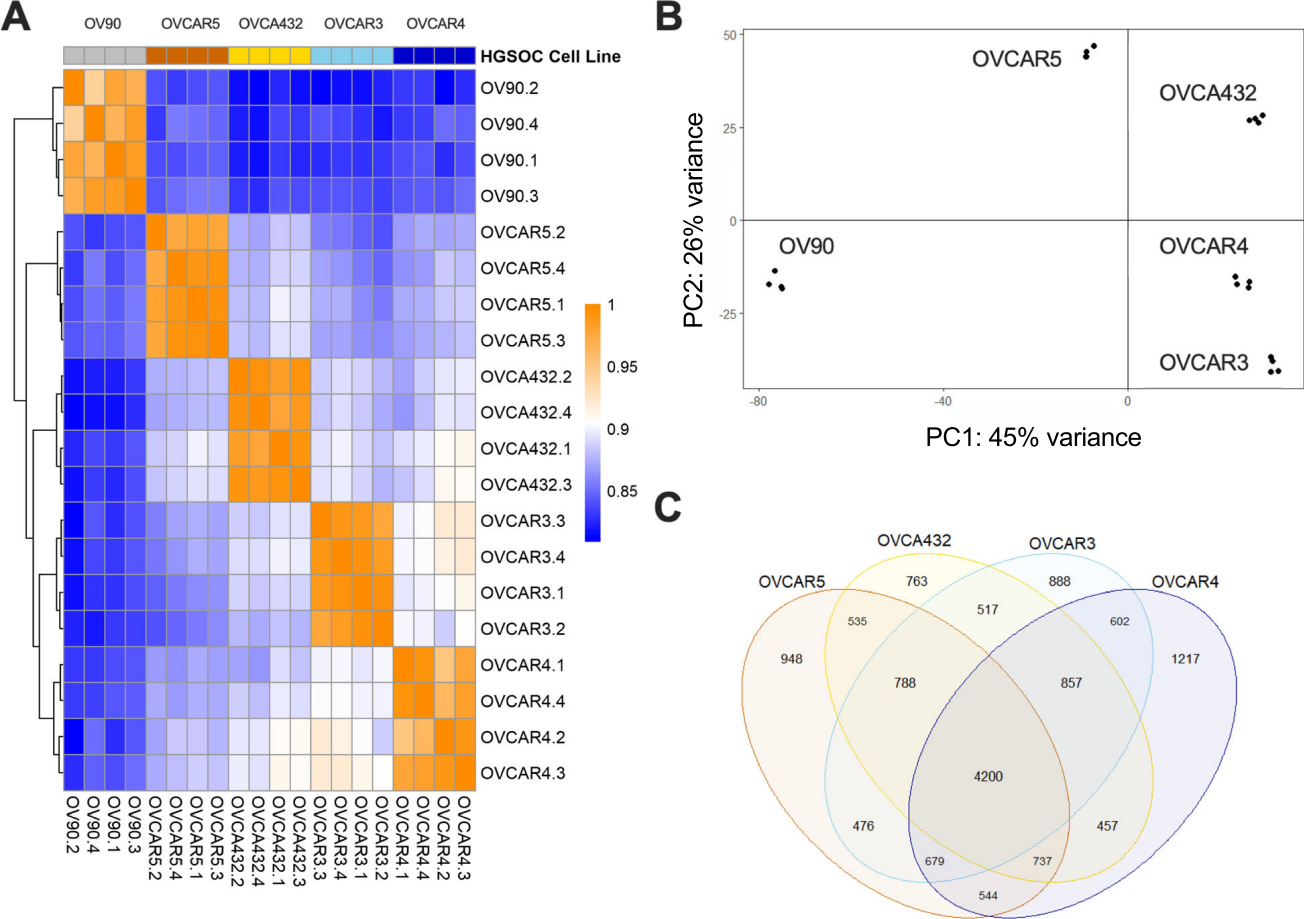
RNA-seq analysis of five HGSOC cell lines revealed global transcriptional heterogeneity. **a** Pearson correlations of gene expression in the HGSOC cell lines OV90, OVCAR3, OVCAR4, OVCAR5, and OVCA432 organized using hierarchal clustering along both rows and columns (*n* = 4). Scale bar represents coefficient of correlation (r). **b** Principal component analysis of variance stabilized expression data based on weighted gene lists. X and Y-axes represent the primary and secondary principal components (PC1, PC2), respectively, and report overall contribution to variance in the dataset. Extension to a third PC captured an additional 17% variance but did not improve interpretation of the data. While PC2 separated the two cell lines with the strongest macrophage-inducing potential (OVCAR5 and OVCA432) from the other lines, this analysis did not identify potential candidates due to the sheer number of genes that had positive loadings in PC2. **c** Number of genes found to be differentially expressed (two-fold up- or down-regulated) in the OVCAR3, OVCAR4, OVCAR5, and OVCA432 cell lines relative to OV90

To identify candidate factors produced by the HGSOC cells that promote macrophage differentiation and AAM polarization, we performed RNA sequencing (RNA-seq) to evaluate the global gene expression profiles of each HGSOC cell line (available here). To analyze this data, we first constructed a heatmap of pairwise Pearson’s correlation coefficients of gene transcription levels (Fig. 3a), performed principal component analysis (Fig. 3b), and evaluated shared gene expression (Fig. 3c). As expected, the replicates for individual cell lines were more similar to each other than to samples from a different line. These analyses demonstrated the heterogeneity across the five HGSOC cell lines, and that OV90 was transcriptionally distinct from the other four HGSOC cell lines. However, the global differences in gene expression across the five HGSOC lines did not follow the same trend as their macrophage/AAM producing-potential and did not reveal which signaling pathways to further investigate. Therefore, we sought to evaluate patterns in the gene expression data beyond differential expression.

**Fig. 4:**
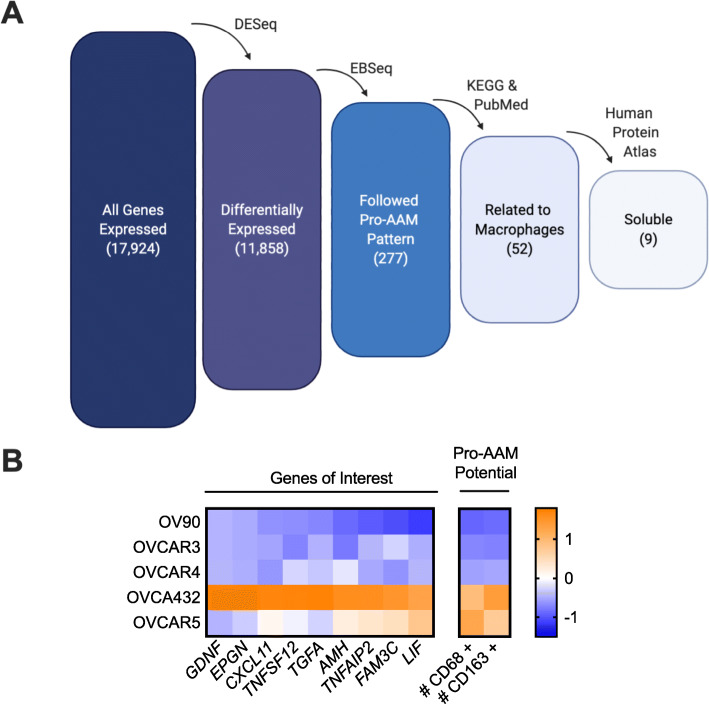
RNA-seq analyzed by EBSeq identified genes whose expression mirrored the pro-AAM potential of the HGSOC cell lines. **a** Diagram of the process by which the nine factors of interest were identified. **b** Gene expression for the nine factors (Z-score normalized) in HGSOC cell lines compared to their pro-AAM potential; values represent the average of 4 microdevices

As a next step we employed EBSeq, an empirical Bayesian method used to identify statistically significant patterns in RNA-seq datasets [22]. EBSeq calculates the probability of a gene being either equal or unequal in a comparison. In the baseline mode, EBSeq does not examine directionality (i.e.*,* identify gene expression patterns of greater than or lesser than between groups). To incorporate directionality, an additional logic gate was added to those that met the EBSeq criteria of belonging to a pattern of interest. Specifically, we looked for two patterns - one that matched the number of CD68+ cells (OV90 < OVCAR4 < OVCAR3 < OVCA432 < OVCAR5) and one that matched the number of CD163+ cells (OV90 < OVCAR4 < OVCAR3 < OVCAR5 < OVCA432. EBSeq was used to determine which genes adhered to the probability OV90 ≠ OVCAR4 ≠ OVCAR3 ≠ OVCAR5 ≠ OVCA432. This subset of genes was then further analyzed for directionality similar to the observed macrophage and AAM potential, OV90 < OVCAR4 < OVCAR3 < OVCAR5 < OVCA432. When applied to the 11,858 differentially expressed genes, EBSeq identified 277 genes as having the same expression pattern as the macrophage/AAM potential (Fig. 4a). To further narrow the list of candidates, we used curated databases to identify candidates with characteristics consistent with our observations. First, KEGG (Kyoto Encyclopedia of Genes and Genomes) Pathway Analysis [43] along with PubMed literature searches were used to identify candidate genes with links to monocyte differentiation or macrophage polarization, or that are secreted by cells in tumor microenvironments or during wound healing, yielding 52 genes of interest. As the cancer cells and MDCs do not contact each other in the device, the Human Protein Atlas (https://www.proteinatlas.org/) [44] was used to identify genes that produce a soluble factor, resulting in a final list of nine genes of interest (Table 1).

**Table 1:** Nine candidate factors identified by EBSeq analysis. Sequencing data is reported as the normalized read counts averaged among technical replicates for each cell line. References refer to previous studies supporting the role of the candidate factor in oncogenesis and/or macrophage behavior

Gene Name	Gene Symbol	OV90	OVCAR4	OVCAR3	OVCAR5	OVCA432	Ref.
Anti-Mullerian Hormone	*AMH*	3.72	4.73	11.70	15.95	30.91	[45, 46]
C-X-C Motif Chemokine Ligand 11	*CXCL11*	0.65	1.06	0.75	3.36	9.22	[47, 48]
Epithelial Mitogen	*EPGN*	0.00	0.00	0.21	2.97	34.75	[49, 50]
Family with Sequence Similarity 3 Member C	*FAM3C*	3410.94	5751.98	4598.91	7852.69	10,933.33	[51]
Glial Cell Line-Derived Neurotrophic Factor	*GDNF*	0.00	0.00	0.16	0.00	47.86	[52]
Leukemia Inhibitory Factor	*LIF*	87.28	586.30	616.71	1515.45	1895.35	[28, 53]
Transforming Growth Factor Alpha	*TGFA*	7.34	586.50	870.77	1035.11	5681.16	[49, 50]
Tumor Necrosis Factor Alpha-Induced Protein 2	*TNFAIP2*	2222.38	3571.95	3406.09	5590.16	8712.42	[54, 55]
Tumor Necrosis Factor Ligand Superfamily Member 12	*TNFSF12*	2.60	0.56	20.68	27.71	104.14	[56-58]

### HGSOC-derived TGFα drives the generation of macrophages

To interrogate select pathways identified from analysis of the RNA-seq data, co-cultures of OVCAR5 and monocytes were treated with small molecule inhibitors or blocking antibodies related to the signaling pathways exploited by the identified candidates. Specifically, family with sequence similarity 3 member C (FAM3C) signaling was inhibited using the γ-secretase inhibitor DAPT [51] and signaling downstream of leukemia inhibitory factor (LIF) and transforming growth factor alpha (TGFα) was inhibited using the selective STAT3 inhibitor galiellalactone (GL) [28]. At the receptor level, signaling downstream of anti-Mullerian hormone (AMH) was inhibited using an antibody against its receptor AMHR2 [59]; and signaling downstream of TGFα and epigen was inhibited using an antibody against the shared receptor, EGFR [17]. Tumor necrosis factor alpha induced protein 2 (TNFAIP2) was not directly interrogated due to lack of specific inhibitors. Tumor necrosis factor superfamily member 12 (TNFSF12)*,* C-X-C motif chemokine ligand 11 (CXCL11) and glial cell derived neurotrophic factor (GDNF) were not directly interrogated since they did not match the pro-AAM pattern as strongly as the other candidates.

**Fig. 5:**
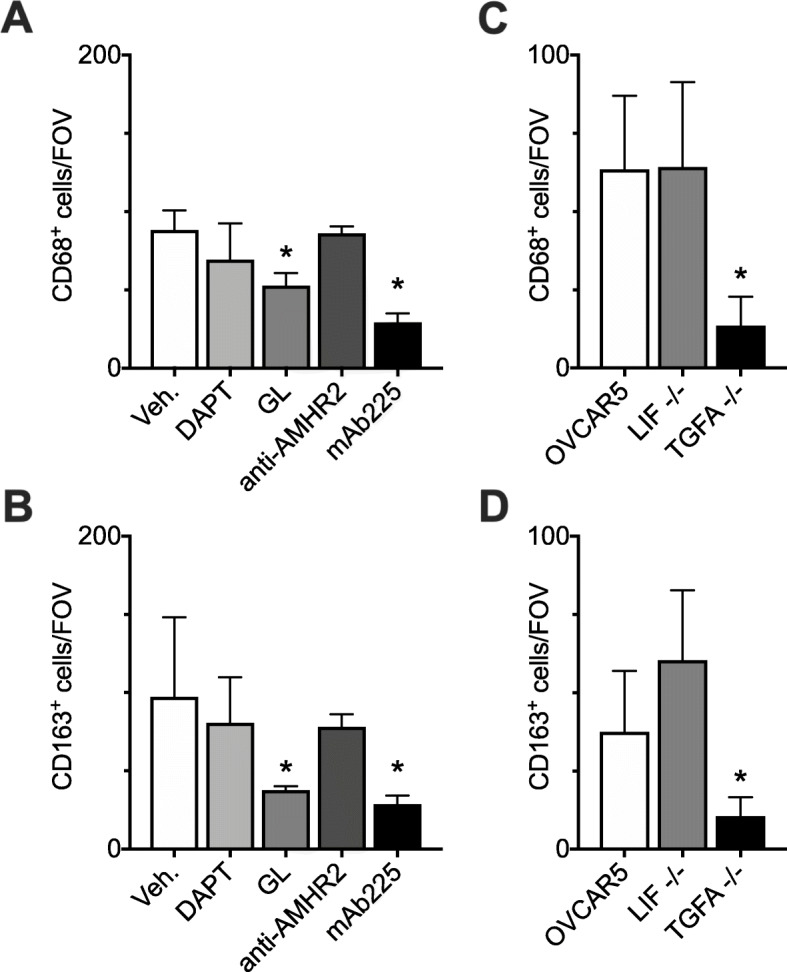
HGSOC-derived TGFα drives the generation of macrophages. **a**, **b** Monocytes were co-cultured with HGSOC cell line OVCAR5 +/− vehicle control (Veh.) or inhibitors against pathways related to the genes of interest: 10 μM DAPT, 5 μM GL, 20 ng/mL anti-AMH, 10 μg/mL mAb225. **a** CD68+ cells per field of view and (**b**) CD163+ cells per field of view. Data expressed as average ± SD, n = 3 microdevices per condition; *p < 0.05 vs. Veh. by one-way ANOVA with Dunnett multiple comparison correction. **c**, **d** Monocytes were co-cultured with HGSOC cell lines OVCAR5, OVCAR5^*LIF−/−*^, or OVCAR5^*TGF −/−*^. **c** CD68+ cells per field of view and (**d**) CD163+ cells per field of view. Data expressed as average ± SD, *n* = 3 microdevices per condition; *p < 0.05 vs. OVCAR5 by one-way ANOVA with Tukey multiple comparison correction

Of the five inhibitors tested, GL and mAb225 significantly reduced the number of CD68+ cells (Fig. 5a) as well as number of CD163+ cells (Fig. 5b) after 7 days of co-culture, suggesting a role for LIF (GL inhibition), TGFα (GL and mAb225 inhibition), or epigen (mAb225). However, since the inhibitors were added to co-culture we could not discern if they were inhibiting HGSOC or MDC derived epigen, TGFα or LIF. Therefore, we next used CRISPR to produce ovarian cancer lines with the specific factor knocked down in OVCAR5. CRISPR attempts in OVCA432 were unsuccessful due to the cell line not tolerating the low densities needed for the protocol (data not shown). Examination of the expression levels in OVCAR5 demonstrated that *TGFA* had significantly higher expression levels compared to *EPGN* (Supplemental Figure 4); therefore we only generated OVCAR5^*TGFA−/−*^ and OVCAR5^*LIF−/−*^. TIDE analysis yielded 84.1 and 87.8% of OVCAR5 cells containing an insertion or deletion mutation at the target site in the *TGFA* or *LIF* loci, with R^2^ = 0.87 and R^2^ = 0.91 respectively (Supplemental Figure 4). At the protein level, the respective CRISPR lines had either non-detectable TGFα or significantly reduced LIF production (Supplemental Figure 4).

Monocytes were then co-cultured with OVCAR5, OVCAR5^*TGFA−/−*^, or OVCAR5^*LIF−/−*^ and the resulting number of CD68+ and CD163+ MDCs were evaluated. Culturing monocytes with OVCAR5^*LIF−/−*^ did not reduce the total number of CD68+ or CD163+ cells per field of view compared to monocytes co-cultured with OVCAR5 (Fig. 5c, d). While LIF is elevated in ascites fluid in ovarian cancer and can promote the generation of AAMs, it does so in an IL-6 and MCSF-dependent manner [53]. Therefore, the fact that we did not see an effect from knocking out LIF may be because the observed macrophage/AAM potential in our system was independent of MCSF. In contrast, culturing monocytes with OVCAR5^*TGFA−/−*^ significantly reduced the total number of both CD68+ and CD163+ cells to approximately 25% of the amount from co-culture with OVCAR5. Similar to our results in baseline co-cultures (Fig. 1), the total number of CD163+ cells closely matched those of the total number of CD68+ cells with either OVCAR5^*LIF−/−*^ or OVCAR5^*TGFA−/−*^, supporting the notion that monocyte to macrophage differentiation is the limiting step in AAM production, rather than AAM polarization. Taken together, these data indicate that TGFα produced by HGSOC cells supports monocyte differentiation into macrophages via EGFR.

## Discussion

TGFα has been previously linked to ovarian cancer progression. For example, TGFα levels have been shown to be elevated in the ascites fluid of patients with stage III/IV ovarian cancer compared to stage I/II [60]. Additionally, histological examinations of the epithelial portion of ovarian tumors [61] and characterization of ovarian cancer lines [62] have shown that some ovarian cancer cells produce TGFα, consistent with our findings here. TGFα has also been shown to be involved in tumor cell/stromal cell interactions in ovarian cancer. For example, in a mouse model of ovarian cancer, tumor cell-derived TNFα induced omental fibroblasts to produce TGFα, which in turn stimulated ovarian cancer EGFR and accelerated metastatic potential [63]. Here we demonstrate that loss of HGSOC-derived TGFα resulted in a significant decrease in the total number of macrophages or AAMs produced in co-culture. These data suggest that TGFα promotes monocyte to macrophage differentiation rather than macrophage to AAM polarization. While the immunomodulatory role of TGFα has not been previously investigated in ovarian cancer, increased TGFα expression has been correlated with increased levels of macrophages in breast cancer. In a recent histological analysis of patients with breast cancer bone metastases, tissue surrounding the tumors exhibited increased macrophage coverage as well as increased TGFα expression compared to the tumor tissue [64]. Additionally, knockdown of *TGFA* in a human breast cancer cell line used in a mouse model of breast cancer decreased the total number of macrophages present in the tumors as well as tumor growth [65]. In vitro, knocking down *TGFA* did not reduce breast cancer cell production of MCSF, but did reduce the expression of CCL2, leading the authors to conclude that breast cancer cell-derived TGFα induced autocrine EGFR signaling, leading to increased CCL2 and macrophage recruitment [65]. In contrast, our findings suggest that cancer cell-derived TGFα can directly support macrophage differentiation.

Epidemiological data support that patients with elevated numbers of tumor associated macrophages have a worse outcome for ovarian cancer as well as other types of cancer [66–68]. Key points where tumor cells have the ability to influence the number of macrophages in solid tumors include the recruitment of circulating monocytes and the differentiation of monocytes to macrophages. Ovarian cancer cells have been shown to express the canonical monocyte recruitment factors, *CCL2* and *CCL7* [69, 70]. In vivo, production of CCL2 by metastatic breast cancer cells has been shown to increase recruitment of inflammatory monocytes [71]. In a similar manner, differentiation can result from tumor cell expression and secretion of the canonical factor, MCSF [38]. Interestingly, our results demonstrated that production of CD68+ macrophages by ovarian cancer cells was independent of MCSF. Recent studies have demonstrated that other mechanisms can support macrophage production, such as secretion of retinoic acid by sarcoma cells that blocks differentiation of monocytes to dendritic cells [72]. To our knowledge, a direct role for TGFα in this process has not been reported.

In addition to influencing macrophage differentiation, prior studies have demonstrated that ovarian cancer cells can direct macrophage polarization to the alternative phenotype [47]. One possible mechanism for this is tumor cell production of hyaluronic acid, which leads to increased cholesterol efflux in macrophages; the resulting alteration in macrophage lipid rafts led to increased gene expression in response to exogenous IL-4 and suppressed gene expression in response to IFN *γ* [73]. A key difference between our work and these studies is that most prior studies of macrophage polarization use monocyte-derived macrophages produced by canonical differentiation with MCSF, rather than tumor cell-directed differentiation [74]. In the paradigm of full differentiation and polarization by tumor cells alone, our results do not support a clear role for TGFα in polarization, as the primary effect of inhibiting TGFα is the loss of CD68+ macrophages rather than a shift in the percentage of CD163+ macrophages.

## Conclusions

Our findings add TGFα as a novel factor that tumor cells can use to directly promote macrophage differentiation. This finding was enabled by a combination of a controlled in vitro co-culture model, bioinformatic analysis of gene expression and phenotype patterns, and rigorous tests of candidate factors identified in our screen. Combined with other studies demonstrating effects of tumor cells on monocyte recruitment, macrophage polarization, and macrophage influence on tumor cells [16–18], it is evident that tumor cell-macrophage interactions are complex and multi-faceted, providing multiple opportunities for intervention to slow tumor progression.

## Supplementary information

**Additional file 1: Supplemental Figure 1.** Ability of the five HGSOC cell lines to produce CD68+ and CD163+ cells compared to the resulting two conditions of interest for EBSeq. (**A**) CD68+ per field of view and (**B**) CD163+ cells per field of view after monocytes have been co-cultured with HGSOC cell lines for seven days. Data are expressed as average ± SD, *n* = 3 monocyte donors. Groups with no statistically significant differences are linked by the same letters, while groups that are statistically different from each other have different letters, *p* < 0.05 by Tukey-HSD. (**D, E**) Conditions of interest for EBSeq that mimic either the number of CD68+ or CD163+ cells after co-culture with HGSOC: *C*1 : *μ*1 = *μ*2 = *μ*3 < *μ*4 < *μ*5; *C*2 : *μ*1 = *μ*2 = *μ*3 < *μ*4 > *μ*5.

**Additional file 2: Supplemental Figure 2.** Immunofluorescent detection of CD68 (a macrophage marker, green) and CD163 (an AAM marker, green) in CAM and AAM controls after seven days of culture. Cells were counter stained with DAPI (blue). Scale bar = 250 μm.

**Additional file 3: Supplemental Figure 3.** (**A**) Gene expression (Z-score normalized) from HGSOC cell lines in monoculture compared to their pro-AAM potential (the resulting number of CD68+ and CD163+ cells after monocytes have been co-cultured with HGSOC cell lines OV90, OVCAR4, OVCAR4, OVCAR5, or OVCA432 for seven days); values represent the average of 4 microdevices. (**B**) Correlation between *TNF* fold change in co-culture relative to OV90 *TNF* expression in co-culture and #CD163+ cells/FOV. (**C**) Cytokine expression (Z-score normalized) from HGSOC cell lines in monoculture compared to their pro-AAM potential; values represent the average of 4 microdevices.

**Additional file 4: Supplemental Figure 4.** (**A,B**) Normalized read counts of (**A**) *EPGN* and (**B**) *TGFA* from RNA-seq analysis of five HGSOC cell lines. Note the difference of scales between *EPGN* and *TGFA*. (**C**) TIDE analysis yielded 87.8% of OVCAR5 cells containing an insertion or deletion mutation at the target site in the *LIF* loci, with R^2^ = 0.91. (**D**) Conditioned media was collected from OVCAR5 and OVCAR5^*LIF −/−*^ after 24 h and analyzed for LIF. Data are expressed as average ± SD, *n* = 4 microdevices per condition; *p < 0.05 vs. by t-test. (**E**) TIDE analysis yielded 84.1% of OVCAR5 cells containing an insertion or deletion mutation at the target site in the *TGFA* loci, with R^2^ = 0.87. (**F**) Conditioned media was collected from OVCAR5 and OVCAR5^*TGFA −/−*^ after 24 h and analyzed for TGFα. Data are expressed as average ± SD, *n* = 4 microdevices per condition; *p < 0.05 by t-test.

## Data Availability

The RNA-seq dataset generated during this study has been deposited on the NCBI sequence read archive (SRA; SUB7817574). The additional data generated during this study are available from the corresponding author on reasonable request.

## Abbreviations

HGSOC: High grade serous ovarian carcinoma
AAM: Alternatively activated macrophage
CAM: Classically activated macrophage
PDMS: Polydimethyl siloxane
PBS: Phosphate buffered saline
MDC: Monocyte derived cell
